# Effects of maternal education on birth preparedness and complication readiness among Ethiopian pregnant women: a systematic review and meta-analysis

**DOI:** 10.1186/s12884-020-2812-7

**Published:** 2020-03-06

**Authors:** Daniel Bekele Ketema, Cheru Tesema Leshargie, Getiye Dejenu Kibret, Moges Agazhe Assemie, Pammla Petrucka, Animut Alebel

**Affiliations:** 1 Department of Public Health, College of Health Science, Debre Markos University, Debre Markos, Ethiopia; 2 Department of Nursing, College of Health Science, Debre Markos University, P.O. Box 269, Debre Markos, Ethiopia; 3 Faculty of Health, University of Technology Sydney, Ultimo, NSW Australia; 4 College of Nursing, University of Saskatchewan, Saskatoon, Canada; 5 School of Life Sciences and Bioengineering, Nelson Mandela African Institute of Science and Technology, Arusha, Tanzania

**Keywords:** Birth preparedness, Complication readiness, Maternal education, Meta-analysis, Systematic review

## Abstract

**Background:**

Birth preparedness and complication readiness are broadly endorsed by governments and international agencies to reduce maternal and neonatal health threats in low income countries. Maternal education is broadly positioned to positively affect the mother’s and her children’s health and nutrition in low income countries. Thus, this systematic review and meta-analysis aims to estimate the effect of maternal education on birth preparedness and complication readiness.

**Methods:**

This review was reported according to the Preferred Reporting Items for Systematic Reviews and Meta-Analysis. We conducted an electronic based search using data bases of PubMed /MEDLINE, Science direct and google scholar. STATA™ Version 14.1 was used to analyze the data, and forest plots were used to present the findings. *I*^*2*^ test statistics and Egger’s test were used to assess heterogeneity and publication bias. Pooled prevalence and pooled odd ratios with 95% confidence intervals were computed. Finally, Duval and Tweedie’s nonparametric trim and fill analysis using random-effects meta-analysis was conducted to account for publication bias.

**Results:**

In this meta-analysis, 20 studies involving 13,744 pregnant women meeting the inclusion criteria were included, of which 15 studies reported effects of maternal education on birth preparedness and complication readiness. Overall estimated level of birth preparedness and complication readiness was 25.2% (95% CI 20.0, 30.6%). This meta-analysis found that maternal education and level of birth preparedness and complication readiness were positively associated. Pregnant mothers whose level of education was primary and above were more likely to prepare for birth and obstetric emergencies (OR = 2.4, 95% CI: 1.9, 3.1) than non-educated mothers.

**Conclusion:**

In Ethiopia, the proportion of women prepared for birth and related complications remained low. Maternal education has a positive effect on the level of birth preparedness and complication readiness. Therefore, it is imperative to launch programs at national and regional levels to uplift women’s educational status to enhance the likelihood of maternal health services utilization.

## Background

In 2015, around 830 women deceased everyday due to pregnancy and childbirth-related complications worldwide. Among these, 550 happened in sub-Saharan Africa and 180 deaths in Southern Asia, compared to 5 in high income countries [1, 2]. Most maternal deaths are attributed to lack of quality maternal care in addition to lack of access to skilled routine emergency care [3, 4].

Most maternal deaths are easily avertable, as health-care solutions to prevent and manage pregnancy related complications are well known. Early antenatal care (ANC), skilled care during childbirth, and care and support in the weeks immediately after birth are the most effective strategies [5]. There is extensive evidence related to delays in deciding, reaching and receiving care and their consequences [6–9]. Although Birth Preparedness or Complication readiness (BPCR) is a broad and integrative strategy, lack of evidence exists in relation to its comprehensive and holistic application. However, components of the BPCR matrix have been employed and assessed in many settings [10–13].

Basic components of birth plan packages include: identification of danger signs, plan for a skilled birth attendant, identifying place of birth, blood donors, and saving money for transport or other costs, should such needs arise [5, 14]. Complications, such as hemorrhage, are common problem and potentially fatal if timely treatment is not obtained [7, 15]. This context makes the BPCR package a key strategy in sub-Saharan Africa (SSA), including Ethiopia, where obstetric services are generally inadequate and/or underutilized. The BPCR initiative was initiated across SSA in the early 2000s for the purpose of increasing health facility births in combination with the introduction of focused antenatal care (FANC) [16, 17].

Ethiopia has a persistently high maternal mortality ratio (MMR), despite progress towards the millennium development goals [18, 19]. Accordingly, in the last 15 years, MMR reduced from 871 to 412 per 100,000 live-births [20]. However, the proportion of BPCR in Ethiopia varied across the regions. Various cross-sectional studies showed that the level of BPCR ranged from as low as 16.5% in Robe District to as high as 56.3% in the Federal Police Referral Hospital, Addis Ababa [17, 21].

Research shows that there is a strong linkage between maternal education and utilization of reproductive health services [22, 23]. When women’s education increases, their own and their children’s health and nutrition are positively impacted [22]. Although literature has shown low maternal education to be a common variable for under or poor practice of BPCR, there is no research to confirm whether it is a consistent finding and the overall effect size has not been established [15, 24, 25]. Hence, this study aims to establish pooled effects of maternal education on BPCR of pregnant women in Ethiopia by quantifying the association between increased maternal education and BPCR practice. Estimating pooled effects of maternal education on BPCR among pregnant mothers is important in addressing birth-related complications as well as for implementing focused ANC. In addition, the purpose of this study was to estimate pooled prevalence of BPCR practice among Ethiopian pregnant women. While it has been estimated and studied in previous research in Ethiopian context [26], limited number of articles were included in the former article [26] and did not address factors affecting for low utilization of BPCR. However, there was a narrative synthesis of qualitative information on implementation of BPCR [27]. But in the motioned study [27] review the existing article using narrative review, which was prone selection and evaluation bias and even not reproducible. Therefore, our study has designed to address these identified gaps. In addition, our study has used more robust design (systematic review and meta-analysis). Moreover, with the growing demand for evidence based interventions of safe motherhood programs, this systematic review and meta-analysis will add to the evidence base of effective promotion and implementation of BPCR.

## Methods

This systematic review has been prepared according to the Preferred Reporting Items for Systematic Review and Meta-Analysis (PRISMA) guideline (Supplementary file 1). We searched the databases: PubMed/MEDLINE, and Science Direct. Google scholar and snowball approach were also employed. The following keywords were used: “level of birth preparedness and complication readiness” [MeSH Terms] OR “birth preparedness and complication readiness” [All fields] AND “birth preparedness” [All fields] OR “complication readiness” [All fields] and “prevalence” [Subheading] OR “pregnant women” [All fields] AND “Ethiopia” [MeSH Terms] OR “Ethiopia” [All fields], “maternal education”.

### Study selection

#### Predefined inclusion criteria

- **Study setting**: Ethiopia.
- **Study participants:** Pregnant women.
- **Publication condition:** All published and unpublished articles.
- **Language:** English language
- **Types of studies:** Observational study designs.
- **Publication date:** Until December 31, 2018

#### Exclusion criteria

- Unable to access full-texts after two email contacts of the principal investigator.

### Outcome of interests

Primary outcome was the pooled prevalence of BPCR among Ethiopian pregnant women. Secondary outcome was the effect of maternal education on the level of BPCR among Ethiopian pregnant mothers. Effect size was estimated in the form of log odds ratios.

### Data collection and quality score

A standardized data extraction format was prepared in the form of Microsoft Excel. This included primary author name, publication year, region, study design, sample size, number of subjects with outcome, prevalence, and study areas. Three reviewers (DBK, AA and GDK) extracted the data independently. Any differences among reviewers were negotiated with review team members until agreement was reached. Two authors (GDK and MAA), using the Newcastle-Ottawa Scale (NOS) quality assessment tool adapted for cross-sectional studies to assess, independently evaluated the quality of each original study [28]. Any disagreements were resolved by taking the mean score. Finally, studies with a scale of ≥5 out of 10 were considered as achieving high quality.

### Heterogeneity and publication bias

Statistical heterogeneity was evaluated using *I*^*2*^ test and *p*-values of Cochrane-Q statistics. Heterogeneity was classified as low, moderate, or high when *I*^*2*^ test statistics results were 25, 50, and 75% [29]. Dispersion of individual results in the forest plots was also used to evaluate heterogeneity visually. To check publication bias, both objective and subjective (funnel plot) methods were used. Mainly, objective methods such as Eggers’ and Beggs’ tests (*p*-value < 0.05) were used to assess publication bias [30, 31]. The result of Eggers’ test revealed statistically significant publication bias (p-value < 0.001). Finally, Duval and Tweedie’s nonparametric trim and fill analysis was performed to account for this publication bias.

### Data synthesis

Relevant data from each study were imported into Stata™ Version 14 for further analysis, and results were presented in Tables and forest plot. A random effects meta-analysis model was employed to estimate the Der Simonian and Laird’s pooled effect because of high levels of heterogeneity. Meta-regression was performed to relate the effects of study characteristics, such as sample size, publication year, and region on pooled estimates of BPCR and identify possible sources of heterogeneity. Sensitivity analysis using a random effects model was performed to assess the influence of a single study on the overall meta-analysis estimate.

## Results

**Fig. 1:**
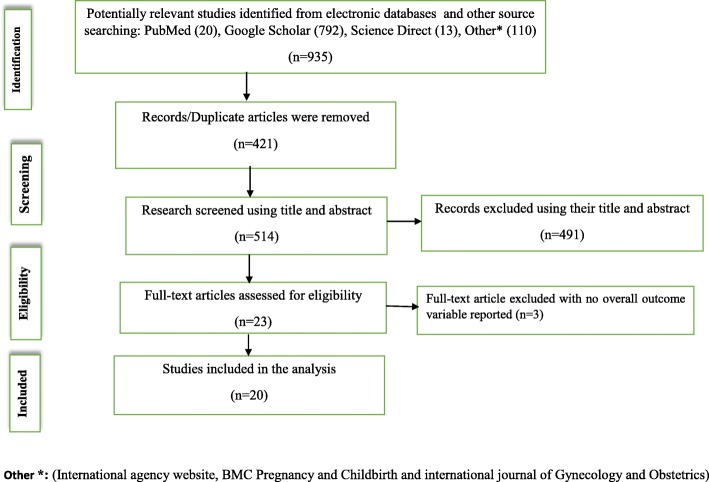
Flow diagram shows the studies selection of the meta-analysis of the effect of maternal education on birth preparedness and complication readiness among pregnant women in Ethiopia

A total of 935 studies were collected from different databases (Fig. 1). All duplicate articles (*n* = 421) were removed. From the remaining 514 articles, 491 articles were excluded because their titles and abstracts were not in line with our inclusion criteria. Lastly, a total of 23 full text studies were downloaded and assessed for eligibility criteria. Among accessed full text articles, three were excluded because in two papers outcome of interests was not reported [32, 33] and one study was not primary study [26]. Final meta-analysis used the remaining 20 studies. The majority (95%) of the studies employed cross-sectional study designs with a total population of 13,744 pregnant women. Prevalence of BPCR ranged from 16.5% in Robe District, Oromia Region [17] to 56.3% in Addis Ababa [21]. Based on the Newcastle-Ottawa Scale for cross-sectional studies quality assessment tool, the quality score ranged from 4 to 9 (Table 1).

**Table 1:** Characteristics of studies included in the systematic review and meta-analysis on the birth preparedness and complication readiness among pregnant mothers in Ethiopia

No	Authors	Year of publication	Design	Sample Size	Study area	BP/CR^a^ [95% CI]	Quality assessment
1	Andarge, E., et al. [34]	2017	Cross-sectional	707	Ariba Minch, SNNPR^b^	30.0 [26.6, 33.4]	7
2	Zepre, K. et al. [25]	2017	Cross-sectional	449	Guraghe Zone, SNNPR	37.0 [33.5, 41.4]	6
3	Mekuaninte A et al. [35]	2016	Cross-sectional	642	Adama Town, Oromia	29.1 [25.6, 32.6]	8
4	Hiluf, M. et al. [36]	2008	Cross-sectional	534	Adigrat, Tigray	22.0 [18.5, 25.5]	6
5	Hailu, M., et al. [8]	2011	Cross-sectional	742	Sidama Zone, SNNPR	17.0 [14.4, 19.6]	7
6	Hailemariam etal [37]	2016	Cross-sectional	356	Debre Birhan, Amhara	53.9 [48.7, 59. 1]	6
7	Gebre, M., et al. [9]	2015	Cross-sectional	569	Wolayta Zone, SNNPR	18.3 [15.2, 21.4]	6
8	Belda, S. et al. [38]	2016	Case-control	358	Goba, Oromia	49.2 [44.0, 54.4]	7
9	Markos, D. et al. [39]	2014	Cross-sectional	580	Goba Woreda, Oromia	29.9 [26.2, 33.6]	6
10	Debelew G. et al. [40]	2014	Cross-sectional	3612	Jimma Zone, Oromia	23.3 [21.9, 24.7]	9
11	Tafa, A., et al. [24]	2018	Cross-sectional	555	Kofale District, Oromia	41.3 [37.2, 45.4]	5
12	Kaso, M. et al. [17]	2014	Cross-sectional	575	Arsi Zone, Oromia	16.5 [13.5, 19.5]	8
13	Iyasu, A., et al. [41]	2018	Cross-sectional	746	Bule Hora, Oromia	27.1 [23.9, 30.3]	7
14	Bitew, Y., et al. [42]	2016	Cross-sectional	819	South Wello, Amhara	24.1 [21.2, 27.0]	7
15	Endeshaw. D. et al. [43]	2018	Cross-sectional	500	Tehuledere, Amhara	44.6 [40.2, 49.0]	8
16	Begashaw, B, et al. [44]	2017	Cross-sectional	392	Mizan Tipe, SNNPR	41.1 [36.2, 46.0]	7
17	Bishaw, W.et al. [45]	2014	Cross-sectional	546	Basoliben, Amhara	26.9 [23.2, 30.6]	8
18	Musa, A. et al. [46]	2016	Cross-sectional	405	Dilchora RH^c^, Dire Dawa	54.7 [49.9, 59.6]	7
19	Tilahun.T. et al. [47]	2016	Cross-sectional	423	Dere Teyara, Harari	42.8 [38.1, 47.5]	8
20	Sebele.T [21]	2015	Cross-sectional	224	FPRH^d^, Addis Ababa	56.3 [49.7, 62.6]	4

### Pooled level of birth preparedness and complication readiness

**Fig. 2:**
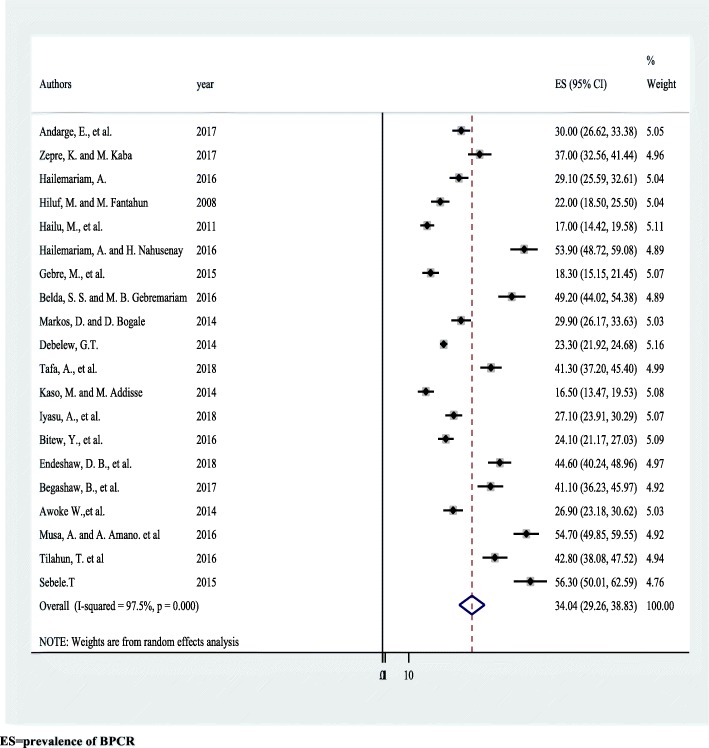
Forest plot displaying the pooled result of birth preparedness and complication readiness among pregnant women in Ethiopia

Overall random effects estimate of the level of BPCR across Ethiopian studies was 34.0% (95% CI: 29.3, 38.8%) (Fig. 2). This observed effect size varies somewhat from study to study. Test statistics results showed high heterogeneity (=97.5%, *p* < 0.001) and Eggers’ test (*p*-value < 0.001) showed significant publication bias. After we applied trim and fill meta-analysis, the overall random effect estimates of BP/CR across studies reduced to 25.2% (95% CI: 20.0, 30.6%) (Table 2). Higher level of BP/CR was observed in Dire Dawa, which was 54.7% (95% CI: 50.0, 62.6%) (Table 3).

**Table 2:** Results from the trim-and-fill method for publication bias in 20 studies on birth preparedness and complication readiness among pregnant women in Ethiopia

Methods	Pooled estimate (%)	95% CI		Z-value	*P*-Value	Estimated Number of studies
		Lower %	Upper %			
Fixed effect	24.2	23.5	24.9	70.1	< 0.001**	27
Random effect	25.2	20.0	30.6	9.2	< 0.001**	

** Significant

**Table 3:** Subgroup level of birth preparedness and complication readiness by region among pregnant women in Ethiopia, 2019

Variable	Characteristics	Included studies	Sample size	Estimate of BPCR (95% CI)
Region	Amhara	4	2221	37.3 (24.1,50.4)
	Oromia	6	6322	31.4 (23.6,39.0)
	SNNPR	6	3615	28.3 (21.0, 35.5)
	Tigray	1	534	22.0 (18.5, 25.5)
	Dire Dawa	1	405	54.7 (49.8, 59.5)
	Harari	1	423	42.8 (38.1, 47.5)
	Addis Ababa	1	224	56.3 (50.0, 62.6)

### Effects of maternal education on BP/CR

**Fig. 3:**
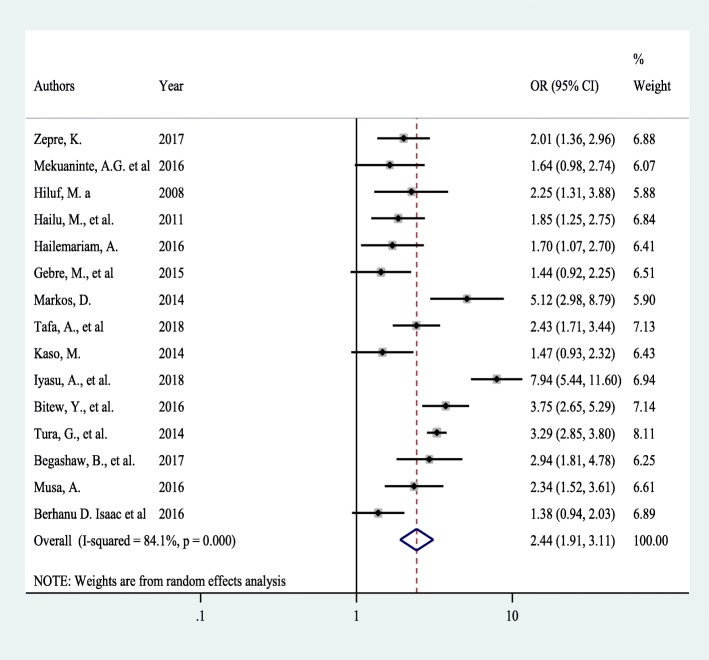
Forest plot of the pooled effect of maternal education on BP/CR practice among pregnant women in Ethiopia

Fifteen studies assessed the effect of maternal education on the prevalence of BPCR. Significant heterogeneity was found across studies (*I*^*2*^ = 84.3%, p < 0.001) which enabled us to use a random effects model. Using this method, our meta-analysis found that maternal education has a significant effect on the BPCR utilization. Pooled odds ratio of BPCR among pregnant women who had primary or greater level of education was 2.44 times more likely as compared to their illiterate counterparts (OR = 2.4, 95% CI: 1.9, 3.1) (Fig. 3).

We explored possible sources of heterogeneity using different statistical techniques. Univariate meta-regression was performed using publication year, sample size, and regions as covariates. However, none of these variables were statistically significant for explaining heterogeneity (Table 4). The presence of publication bias was also assessed using funnel plot and Eggers’ and Beggs’ statistical tests at 5% significant level. The funnel plot shows a symmetrical distribution (Supplementary file 2). However, the Beggs’ and Egger tests showed no significant publication bias with *p*-values of 0.6 and 0.5 respectively. Therefore, publication bias is not a problem.

**Table 4:** Related factors with heterogeneity of the effects of maternal education on BPCR among pregnant women from 2001 to 2018

Variables	Coefficients	*P*-value
Publication year	0.0887168	0.751
Sample size	0. 001196	0.651
Region	0.0517334	0.644

### Sensitivity analysis

To detect the influence of one study on the overall meta-analysis estimate, sensitivity analysis using a random effects model did not show strong evidence for the influence of a single study on the overall result (Supplementary file 3).

## Discussion

Overall pooled estimate of the prevalence of BPCR across Ethiopian studies was 34.0% (95% CI: 29.3, 38.8%); this finding, however, has been affected by publication biases. To account for this, the trim and fill meta-analysis found that only 25.2% (95% CI: 20.0, 30.6%) were well prepared for birth and related complications by practicing elements of BPCR. Our finding is higher than studies done in the rural Gambia (14%) and Kenya (6.9%) [48, 49]. Discrepancies may be due to Ethiopia’s flexible initiatives over time to bring more attention to the issue. On the contrary, however, our finding is much lower than in the rural parts of Uganda (35%), Nigeria (61%), Central Tanzania (58.20%), West Bengal, India (57%), Indore City, India (47.8%), and Thailand (78.6%) [13, 50–54].

These differences could be due to socio-cultural issues, like the use of traditional birth attendants, women’s educational and economic status, and differences in the quality of antenatal care services. Participation of non-governmental organizations (NGOs), which may introduce safe motherhood and female rights, may differ in different parts of these countries. Moreover, this finding is slightly lower than a previous meta-analysis from Ethiopia using 13 studies (32%) [26]. This discrepancy could be due to less studies (13) in the previous review, whereas we included 20 studies. Smaller number of studies may overestimate results. This finding has important implications and should alarm relevant stakeholders to invest in health promotion with regard to BPCR, at all stages of women’ reproductive lives, with support from health care workers. In addition, quality and methods of antenatal care education, including evaluation of how women are benefitted from such education, have to be re-assessed.

Maternal education has a significant effect on the prevalence of BPCR (OR = 2.4, 95% CI: 1.9, 3.1). This is in agreement with studies conducted in southern Tanzania and India [52, 55, 56]. Educated women have better access to information and an enhanced position of women in the household may lead to enhanced decision-making power with regard to health-related issues. Women with formal education were found more likely to have knowledge on the key components of birth preparedness and complication readiness as compared to uneducated women. In a qualitative study on BPCR in rural Tanzania educated women were more likely to accept BPCR-elements, to be better informed, to make healthier choices and more likely to develop and implement a birth plan, and to be more socially or financially authorized to make the required decisions in case of obstetric emergencies [23].

Ethiopia has made significant progress towards reducing maternal and neonatal deaths through BPCR, but progression is relatively unsatisfactory. Therefore, it is recommended to launch programs at national and regional level to raise women’s educational status, which would enhance utilization of maternal health services. Another expected effect of maternal education is to increase BPCR knowledge, which may change individual attitudes. Literate women know about danger signs or possibility of obstetric complications as well as about the importance of using skilled birth attendants. Health workers or volunteers should include literate women in meetings of female clusters to share their experiences with uneducated women. Symbolic cards, video-films, and case stories can be used during prenatal meetings to promote understanding of BPCR messages. Knowledge on birth preparedness and key danger signs in pregnancy, childbirth and postpartum are found to be a significant influencer on utilization of skilled attendants [7, 57, 58].

## Limitations of the study

Tremendous efforts have been made to include all papers from Ethiopia, but only articles published in English were considered. Moreover, 95% of the studies in this meta-analysis employed a cross-sectional study design. Cause-effect relationships, therefore, cannot be shown in this review. Finally, we were unable to get studies from Benishangul Gumuz, Ethio-Somali, Afar, and Gambella regions and this affects generalizability.

## Conclusion

In Ethiopia, the proportion of women who are birth prepared and ready for complications remained low. Pregnant women with primary and higher levels of education were better prepared for birth and related complications than uneducated counterparts. Therefore, it is imperative to launch sustainable programs at national and regional levels which uplift women’s educational status to enhance utilization of maternal health services.

## Supplementary information

**Additional file 1.** PRISMA 2009 checklist.

**Additional file 2.** Funnel plot of on effect of maternal education on BPCR among pregnant women in Ethiopia.

**Additional file 3.** Sensitivity analysis for single study influence on the overall meta-analysis estimate of effect of maternal education on BPCR among pregnant women in Ethiopia.

## Data Availability

Data will be available from the corresponding author upon resendable request.

## Abbreviations

BPCR: Birth preparedness and complication readiness
CI: Confidence interval
FANC: Focused antenatal care
MMR: Maternal mortality ratio
PRISMA: Preferred reporting items for systematic reviews and meta-analyses
SE: Standard error
SNNPR: South Nation and Nationalities People of the Region
WHO: World Health Organization
