# Supplementary figures and images for: Effects of maternal education on birth preparedness and complication readiness among Ethiopian pregnant women: a systematic review and meta-analysis
Source: BMC Pregnancy Childbirth. 2020 Mar 6;20:149. doi: 10.1186/s12884-020-2812-7 (PMC7060625; doi:10.1186/s12884-020-2812-7)

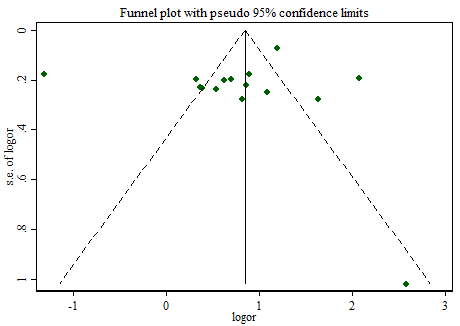

Supplement: Supplementary file 2 — Additional file 2. Funnel plot of on effect of maternal education on BPCR among pregnant women in Ethiopia. [file 12884_2020_2812_MOESM2_ESM.docx]
