# Supplementary material for: Effects of maternal education on birth preparedness and complication readiness among Ethiopian pregnant women: a systematic review and meta-analysis
Source: BMC Pregnancy Childbirth. 2020 Mar 6;20:149. doi: 10.1186/s12884-020-2812-7 (PMC7060625; doi:10.1186/s12884-020-2812-7)
Supplement: Supplementary file 3 [file 12884_2020_2812_MOESM3_ESM.docx]

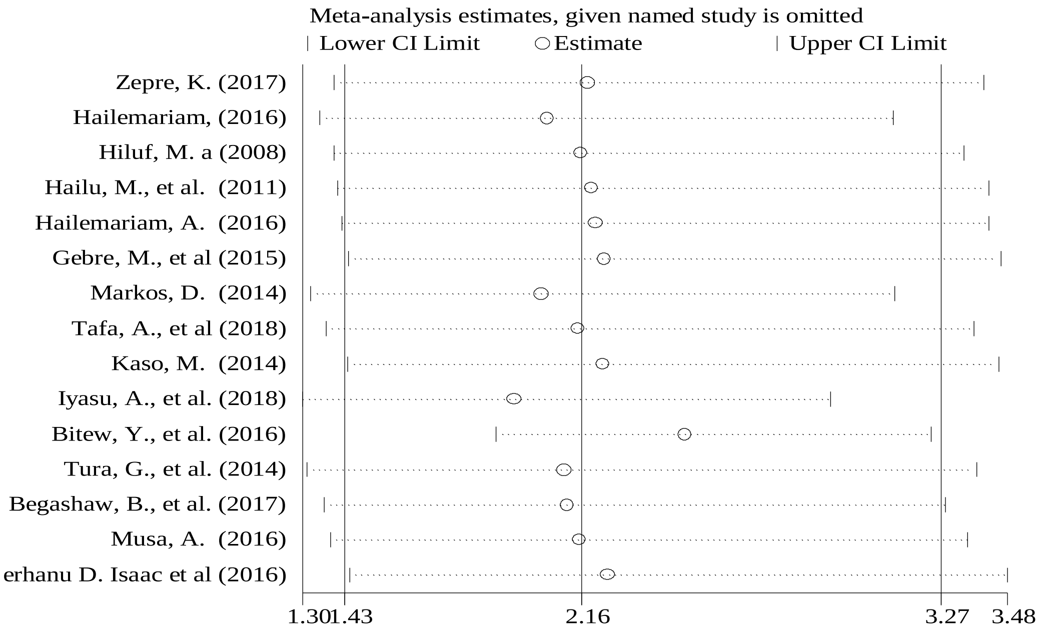
Meta-analysis estimates, given named study is omitted
Lower CI Limit
Estimate
Upper CI Limit
Zepre, K. (2017)
Hailemariam, (2016)
Hiluf, M. a (2008)
Hailu, M., et al. (2011)
Hailemariam, A. (2016)
Gebre, M., et al (2015)
Markos, D. (2014)
Tafa, A., et al (2018)
Kaso, M. (2014)
Iyasu, A., et al. (2018)
Bitew, Y., et al. (2016)
Tura, G., et al. (2014)
Begashaw, B., et al. (2017)
Musa, A. (2016)
erhanu D. Isaac et al (2016)
1.30
1.43
2.16
3.27
3.48
